# Supplementary material for: Is Right Angular Gyrus Involved in the Metric Component of the Mental Body Representation in Touch and Vision? A tDCS Study
Source: Brain Sci. 2021 Feb 25;11(3):284. doi: 10.3390/brainsci11030284 (PMC7996561; doi:10.3390/brainsci11030284)
Supplement: Supplementary file 1 [file brainsci-11-00284-s001.pdf]

Calibration of Visual stimuli

An ad hoc study was conducted to fine-tune the visual stimuli. 24 right-handed subjects, including 8 males, aged 23 to 30 years (25.6 ± 1.9) took part in this study.

Each subject was administered a task divided into two different conditions: one aimed at detecting the ability to discriminate distances between visual stimuli projected onto the body (experimental condition) and one aimed at detecting the ability to discriminate visual stimuli projected into space outside the body, such as the desk (control condition).

Stimuli and task

The experimental paradigm consisted of two pairs of visual stimuli, projected in rapid succession (stimulus duration 100 msec, interstimulus duration 150 msec) in two distinct areas of the body (arm and hand) and in two distinct areas of the desk placed in front of the subject (top and bottom), depending on the task presented.

Following the presentation of the two pairs of visual stimuli, subjects were required to answer the question <<Where do you perceive the greatest distance between pairs of dots?>>. Depending on the test administered (body or desk), the possible responses were "arm" or "hand", in the experimental condition, and "up" or "down", in the control condition.

The visual stimuli were projected through 8 small lasers capable of producing small dots of about 1.5 mm in diameter. 4 lasers (full diameter of the device = 1 cm) projected onto the hand (or desk-down), 4 projected onto the arm (or desk-up). The lasers were applied on an articulated arm that allowed to adjust the distance of the two rows of lasers according to the size of the subject's arm. In addition, the lasers were placed on a mobile support that allowed them to be oriented with respect to the size of the subject's limb. This feature of the set-up is very important because each hand and each forearm were of different sizes and to project the same distances reliably there was a need to orient each laser in the correct positions (see figure below 1S)

Figure 1. S. Experimental set-up.

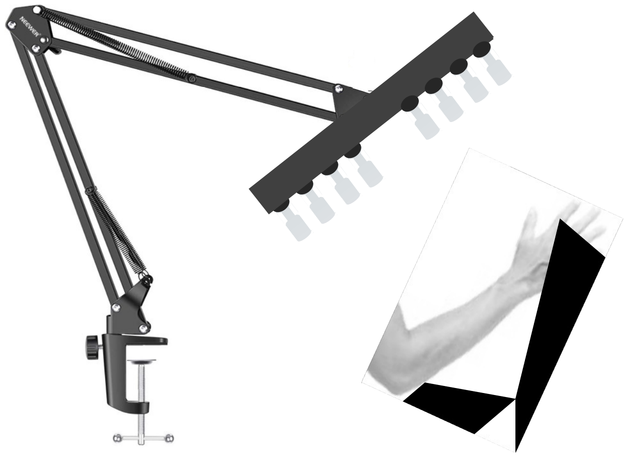

The lasers were connected to a computer that managed the sequence of trails and recorded voice responses.

Each trial consisted of 4 blocks (2 per condition: experimental and control) that were randomized among subjects in order to limit possible bias due to the order of presentation of the sequences. The choice of the division into blocks has the aim of reducing as much as possible any effect due to subject fatigue.

The first pair of stimuli could present a distance equal to 0.2, 0.4, 0.6 or 0.8 cm, the second pair of laser dots could correspond to a greater, equal or lesser distance compared to the first pair. From the different comparisons, we identified 7 different levels of difficulty which were used for the elaboration of the psychophysical curves, as described below. The comparisons ± 3 correspond to the

"easy" level, whereas ± 1 correspond to the "difficult" level, as it is assumed that a smaller distance between the two pairs of stimuli corresponds to a greater discrimination difficulty. ± 2 represent a medium level of difficulty

Each block is composed of 84 trials, 12 for each difficulty level (-3, -2, -1, 0, +1, +2, +3), for a total of 240 trials per subject. The dependent variables taken into account for the analysis of the data are vocal response times, expressed in milliseconds, and the subjects' accuracy, expressed in terms of the percentage of correct answers out of the total answers given by the subject according to the level of difficulty.

Results

Figure below shows the psychophysical relationship between physical distance and perceived stimulus distance (greatest distance between pairs of dots).

Figure 2. S. Psychophisical results.

Y axis shows the percentage of trials (group average) in which subjects judged the changing stimulus as bigger as a function of its difficulty X axis represents the difficulty (±3 easy trials; ±2 = medium trials, ±1 = difficult trials, 0 = no difference)

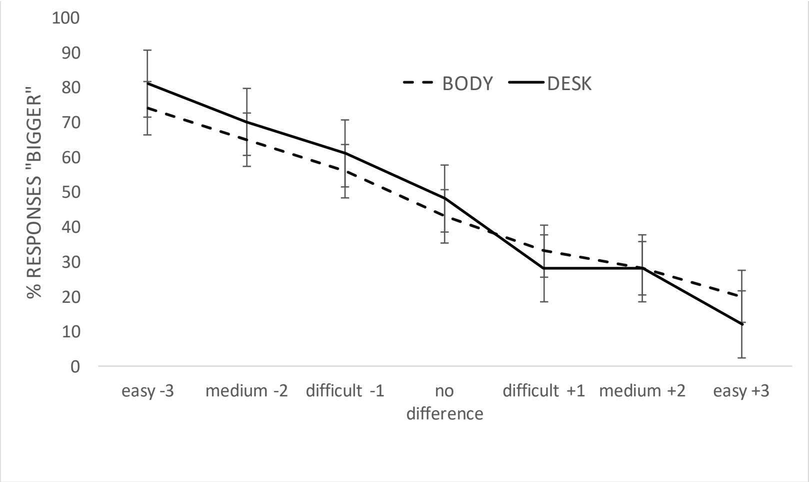

Table S1. Bonferroni post-hoc comparisons following Anova on RTs (ms) of experiment 1. Mean values in brackets.

	**Easy (596.5)**	**Medium (676.7)**	**Difficult (722.1)**
**Easy (596.5)**	---	p=0.037	p=0.003
**Medium (676.7)**	p=0.037	---	p=0.043
**Difficult (722.1)**	p=0.003	p=0.043	---
